# Statins Impair Antitumor Effects of Rituximab by Inducing Conformational Changes of CD20

**DOI:** 10.1371/journal.pmed.0050064

**Published:** 2008-03-25

**Authors:** Magdalena Winiarska, Jacek Bil, Ewa Wilczek, Grzegorz M Wilczynski, Malgorzata Lekka, Patrick J Engelberts, Wendy J. M Mackus, Elzbieta Gorska, Lukasz Bojarski, Tomasz Stoklosa, Dominika Nowis, Zuzanna Kurzaj, Marcin Makowski, Eliza Glodkowska, Tadeusz Issat, Piotr Mrowka, Witold Lasek, Anna Dabrowska-Iwanicka, Grzegorz W Basak, Maria Wasik, Krzysztof Warzocha, Maciej Sinski, Zbigniew Gaciong, Marek Jakobisiak, Paul W. H. I Parren, Jakub Golab

**Affiliations:** 1 Department of Immunology, Center of Biostructure Research, the Medical University of Warsaw, Warsaw, Poland; 2 Department of Laboratory Diagnostics and Clinical Immunology, the Medical University of Warsaw, Warsaw, Poland; 3 Department of Pathology, Center of Biostructure Research, the Medical University of Warsaw, Warsaw, Poland; 4 Laboratory of Molecular and Systemic Neuromorphology, Nencki Institute of Experimental Biology, Warsaw, Poland; 5 Department of Applied Spectroscopy, The Henryk Niewodniczanski Institute of Nuclear Physics, Krakow, Poland; 6 Genmab, Utrecht, The Netherlands; 7 Laboratory of Neurodegeneration, International Institute of Molecular and Cell Biology, Warsaw, Poland; 8 Department of Lymphoproliferative Disease, Maria Sklodowska-Curie Memorial Cancer Center, Institute of Oncology, Warsaw, Poland; 9 Department of Hematology, Oncology and Internal Diseases, the Medical University of Warsaw, Warsaw, Poland; 10 Institute of Hematology and Transfusion Medicine, Warsaw, Poland; 11 Department of Internal Diseases, Hypertension and Vascular Disease, the Medical University of Warsaw, Warsaw, Poland; Baylor College of Medicine, United States of America

## Abstract

**Background:**

Rituximab is used in the treatment of CD20^+^ B cell lymphomas and other B cell lymphoproliferative disorders. Its clinical efficacy might be further improved by combinations with other drugs such as statins that inhibit cholesterol synthesis and show promising antilymphoma effects. The objective of this study was to evaluate the influence of statins on rituximab-induced killing of B cell lymphomas.

**Methods and Findings:**

Complement-dependent cytotoxicity (CDC) was assessed by MTT and Alamar blue assays as well as trypan blue staining, and antibody-dependent cellular cytotoxicity (ADCC) was assessed by a ^51^Cr release assay. Statins were found to significantly decrease rituximab-mediated CDC and ADCC of B cell lymphoma cells. Incubation of B cell lymphoma cells with statins decreased CD20 immunostaining in flow cytometry studies but did not affect total cellular levels of CD20 as measured with RT-PCR and Western blotting. Similar effects are exerted by other cholesterol-depleting agents (methyl-β-cyclodextrin and berberine), but not filipin III, indicating that the presence of plasma membrane cholesterol and not lipid rafts is required for rituximab-mediated CDC. Immunofluorescence microscopy using double staining with monoclonal antibodies (mAbs) directed against a conformational epitope and a linear cytoplasmic epitope revealed that CD20 is present in the plasma membrane in comparable amounts in control and statin-treated cells. Atomic force microscopy and limited proteolysis indicated that statins, through cholesterol depletion, induce conformational changes in CD20 that result in impaired binding of anti-CD20 mAb. An in vivo reduction of cholesterol induced by short-term treatment of five patients with hypercholesterolemia with atorvastatin resulted in reduced anti-CD20 binding to freshly isolated B cells.

**Conclusions:**

Statins were shown to interfere with both detection of CD20 and antilymphoma activity of rituximab. These studies have significant clinical implications, as impaired binding of mAbs to conformational epitopes of CD20 elicited by statins could delay diagnosis, postpone effective treatment, or impair anti-lymphoma activity of rituximab.

## Abstract 2

Jakub Golab and colleagues found that statins significantly decrease rituximab-mediated complement-dependent cytotoxicity and antibody-dependent cellular cytotoxicity against B cell lymphoma cells.

## Editors' Summary

**Background.:**

Lymphomas are common cancers of the lymphatic system, the tissues and organs that produce and store the white blood cells (lymphocytes) that fight infections. In healthy people, the cells in the lymph nodes (collections of lymphocytes in the armpit, groin, and neck) and other lymphatic organs divide to form new cells only when the body needs them. Lymphomas form when a T or B lymphocyte starts to divide uncontrollably. The first sign of lymphoma is often a painless swelling in the armpit, groin, or neck caused by lymphocyte overgrowth in a lymph node. Eventually, the abnormal (malignant) lymphocytes, which provide no protection against infectious diseases, spread throughout the body. Treatments for lymphoma include chemotherapy (drugs that kill rapidly dividing cells) and radiotherapy. In addition, a drug called rituximab was recently developed for the treatment of some types of B cell lymphoma. Rituximab is a monoclonal antibody, a laboratory-produced protein. It binds to a protein called CD20 that is present on the surface of both normal and malignant B lymphocytes and induces cell killing through processes called “complement-dependent cytotoxity” (CDC) and “antibody-dependent cellular cytotoxity” (ADCC).

**Why Was This Study Done?:**

Although rituximab lengthens the lives of patients with some types of B cell lymphoma, it is not a cure—the lymphoma usually recurs. Researchers are trying to increase the effectiveness of rituximab by combining it with other anticancer agents. One group of drugs that might be combined with rituximab is the “statins,” drugs that reduce the risk of heart disease by lowering the level of cholesterol (a type of fat) in the blood. In laboratory experiments, statins kill some cancer cells, in part by altering the fat composition of their outer (plasma) membrane. In addition, some population-based studies suggest that statin treatment might slightly decrease the risk of developing some kinds of cancer, including lymphoma. Statins are already undergoing clinical evaluation in combination with chemotherapy for the treatment of lymphoma, but in this study, the researchers investigate the influence of statins on rituximab-induced killing of B cell lymphomas.

**What Did the Researchers Do and Find?:**

When the researchers tested the ability of rituximab and statin combinations to kill B cell lymphoma cells growing in dishes, they found that statins decreased rituximab-dependent CDC and ADCC of these cells. Statin treatment, they report, did not alter the total amount of CD20 made by the lymphoma cells or the amount of CD20 in their plasma membranes, but it did reduce the binding of another anti-CDC20 monoclonal antibody to the cells. Because both this antibody and rituximab bind to a specific three-dimensional structure in CD20 (a “conformational epitope”), the researchers hypothesized that statins might alter rituximab-induced killing by affecting the shape of the CD20 molecule on the lymphoma cell surface. To test this idea, they used two techniques—atomic force microscopy and limited proteolysis. The data obtained using both approaches confirmed that statins induce shape changes in CD20. Finally, the researchers took B cells from five patients who had taken statins for a short time and showed that this treatment had reduced the amount of anti-CD20 monoclonal antibody able to bind to these cells.

**What Do These Findings Mean?:**

These findings indicate that statins change the shape of the CD20 molecules on the surface of normal and malignant B lymphocytes, probably by changing the amount of cholesterol in the cell membrane. This effect of statins has several clinical implications, which means that cancer specialists should check whether patients with known or suspected B cell lymphoma are taking statins to treat high cholesterol. First, the impaired binding of monoclonal antibodies to conformational epitopes of CD20 in patients being treated with statins might delay the diagnosis of B cell lymphomas (CD20 binding to lymphocytes is used during the diagnosis of lymphomas). Second, some patients with B cell lymphoma may receive an incorrect diagnosis and may not be offered rituximab. Finally, because statins impair the anti-lymphoma activity of rituximab, a possibility that needs to be investigated in clinical studies, cancer specialists should check that patients with B cell lymphoma are not taking statins before prescribing rituximab.

**Additional Information.:**

Please access these Web sites via the online version of this summary at http://dx.doi.org/10.1371/journal.pmed.0050064.

- The MedlinePlus has an encyclopedia page on lymphoma and a list of links to other sources of information on lymphoma (in English and Spanish)
- The US National Cancer Institute provides information about lymphoma and about statins and cancer prevention (in English and Spanish)
- The UK charity Cancerbackup provides information for patients and caregivers on different types of B-cell lymphoma and on rituximab
- The US Leukemia and Lymphoma Society also provides information for patients and caregivers about lymphoma

## Introduction

Rituximab, a chimeric IgG1 monoclonal antibody (mAb) that binds the CD20 antigen, is approved for first-line treatment of follicular and diffuse large B cell lymphoma in combination with chemotherapy, and for the treatment of rheumatoid arthritis [1–3]. Anti-CD20 mAbs have also shown promising therapeutic activity in other cancer and autoimmune indications such as chronic lymphocytic leukemia, Waldenström's macroglobulinemia, systemic lupus erythematosus, and idiopathic thrombocytopenic purpura [3–5]. Although rituximab has revolutionized the treatment of various forms of B cell lymphomas, virtually all patients experience a relapse after single-agent treatment [6]. Novel treatment regimens in which rituximab is combined with other anticancer agents are therefore studied intensively to increase antitumor activity. Combination treatments that benefit from the good safety profile of rituximab are needed especially for elderly patients who poorly tolerate more intensive therapies.

One group of therapeutics for potential use in the combination with rituximab includes competitive inhibitors of 3-hydroxy-3-methylglutaryl-coenzyme A reductase (HMG-CoAR), referred to as statins. Due to their ability to lower blood cholesterol levels, statins have been commonly used in elderly patients to prevent and to treat atherosclerosis of the coronary vessels [7]. HMG-CoAR is the rate-limiting enzyme of the mevalonate pathway essential for the synthesis of isoprenoid compounds including cholesterol, dolichol, and ubiquinone. Isoprenoid prenyl groups (farnesyl- and geranylgeranyl pyrophosphate) are essential for posttranslational modification of numerous cellular proteins, including Ras and Rho family members [8]. Prenylation of these proteins is required for correct membrane localization and their participation in various signaling pathways. By impairing protein prenylation, statins exert significant cytostatic and cytotoxic effects against many solid tumor cell lines as well as hematological malignancies [9]. Some of the antitumor effects of statins might also result from the ability of these drugs to interfere with the formation of cholesterol-rich microdomains within the plasma membrane, commonly referred to as lipid rafts [10,11]. Importantly, statins were shown to exert antilymphoma effects in vitro [12,13] and in vivo [10,14]. A recent European multicenter case-control study EPILYMPH revealed that statin use is associated with a reduction in lymphoma risk in humans [15], although these results are at odds with studies carried out in Japan [16]. High-dose simvastatin was safe and tolerable when combined with chemotherapy in patients with relapsed or refractory myeloma or lymphoma [17], and several statins are now undergoing clinical evaluation in patients with various types of lymphomas. CD20, the target for rituximab, is a non-glycosylated integral membrane protein that is expressed on the surface of virtually all normal and malignant B cells. CD20 represents an attractive therapeutic antibody target, as it does not undergo shedding or internalization and neither a circulating isoform nor an endogenous ligand exist that might interfere with antibody binding. The antitumor effects of rituximab include complement-mediated lysis, antibody-dependent cellular cytotoxicity (ADCC), induction of apoptosis, and potential delayed “vaccine” effects resulting from the targeting of tumor-derived antigens from damaged tumor cells to antigen-presenting cells [18,19]. Rituximab has been shown to redistribute CD20 to lipid rafts, which are liquid-ordered domains of the plasma membrane composed of lipid aggregates with increased cholesterol content [20]. Rituximab-mediated segregation of CD20 into lipid rafts correlates with complement-mediated lysis of target cells [21,22]. The integrity of lipid rafts seems to play a crucial role for a CD20-induced calcium influx and induction of apoptosis [23]. Cholesterol depletion with methyl-β-cyclodextrin (MβCD) profoundly reduces apoptosis [23] as well as src family kinase-dependent calcium mobilization [24] induced by rituximab-mediated crosslinking of CD20. Based on the potential convergence of lipid-raft–dependent antitumor effects of rituximab with the lipid-modulating antitumor activity of statins, we decided to evaluate the influence of statins on rituximab-mediated cytotoxicity.

## Methods

### Cell Culture

The Burkitt's lymphoma cell lines Raji, Ramos, and Daudi, obtained from the American Tissue Culture Collection, were cultured in RPMI 1640 (Sigma Aldrich) supplemented with 10% heat-inactivated FBS, 100 μg/ml streptomycin, and 250 ng/ml amphoterycin B (Gibco Invitrogen). The cells were cultured at 37 °C in 5% CO_2_ in a humidified atmosphere and passaged approximately every other day.

### Leukocyte Isolation from Blood and Bone Marrow

Peripheral blood (15 ml) of five patients was taken before (day 0) or 3 d after atorvastatin (80 mg/d) treatment. Bone marrow (10 ml) of four patients with B lymphoma was aspirated from the hip to the heparinized tube. Peripheral blood or bone marrow was diluted twice with PBS (final volume, 20 ml). Next, 3 ml of Histopaque-1077 (Sigma Aldrich) was pipetted into two conical centrifuge tubes. A total of 10 ml of diluted peripheral blood or bone marrow was slowly layered on the top of Histopaque layer (bone marrow floated on top of Histopaque layer). Probes were centrifuged (400*g* for 15 minutes at 25 °C) without brake. The white blood cell ring and plasma were isolated and washed twice with PBS. The leukocyte pellet was resuspeneded in 5 ml of medium (RPMI or OptiMEM) and counted in a Bürker chamber using Türk dye. Peripheral blood leukocytes were used for the determination of CD20/CD21 ratio (described below), and bone marrow cells were used for experiments with MβCD (as described below for Raji cells) or frozen in freezing medium (500 μl medium, 500 μl of FBS, and 100 μl of DMSO) at −80 °C.

The five patients recruited for the in vivo study had hypercholesterolemia, but were otherwise healthy volunteers who were not statin users. Approval for the study was obtained from the Institutional Review Board of the Medical University of Warsaw and was conducted according to the Declaration of Helsinki. Each patient signed a written informed consent for the procedures.

### Reagents

The following statins were used: atorvastatin (Pfizer Pharmaceuticals), cerivastatin (Bayer), mevastatin (ICN Biochemicals), simvastatin and lovastatin (both from Merck, Sharp & Dohme Research Laboratory), pravastatin (Bristol-Myers Squibb), and fluvastatin (Novartis Pharma). Mevalonic acid, berberine chloride, filipin III, and MβCD were purchased from Sigma Aldrich. Farnesyltransferase-277 (FTI-277) and geranylgeranyltransferase-298 (GGTI-298) were from Calbiochem. Water-soluble cholesterol was purchased from ICN Biochemicals. Rituximab, chimeric IgG,1 was purchased from Roche. Ofatumumab (2F2; HuMax-CD20) is a fully human IgG1. B1, mouse IgG2a, was purchased from Coulter.

### Cell Viability Assays

An assay with 3-[4,5-dimethylthiazol-2-yl]-2,5-diphenyltetrazolium bromide (MTT) reduction was performed as described [25]. Cells (control or drug-treated), rinsed twice with PBS, were seeded into a 96-well flat bottom plate in FBS-free medium (1 × 10^5^/well in 50 μl). Rituximab (10 μg/ml) and human AB serum (10% final concentration; as a complement source) were added to a final volume of 100 μl/well. After a 1-h incubation, MTT solution (5 mg/ml) was added to each well. A 4-h incubation at 37 °C was stopped by the addition of 100 μl of 20% SDS in a mixture of *N*,*N*-dimethylformamide and distilled water (1:2, v/v). The absorbances of the samples were measured on a microplate reader (Bio-Rad Model 680 XR) at 570 nm after overnight incubation at 37 °C. Cell viability was expressed as a relative viability of tumor cells (% of control cultures incubated with medium only) and was calculated as follows: relative viability = [(*Ae* − *Ab*) / (*Ac* − *Ab*)] × 100, where *Ab* is the background absorbance, *Ae* is the experimental absorbance, and *Ac* is the absorbance of untreated controls.

For the trypan blue exclusion test, control and lovastatin pretreated cells were collected, and viable cells were counted in a Bürker chamber using Türk dye. The percentage of treated viable (unstained) cells as compared to viable cells was determined as a percentage of viability.

Alamar blue (Biosource International) absorbance was measured on a fluorescent microplate reader (FLUOStar Optima; BMG Labtech). The change in the color of the culture medium was monitored by measuring A_540_ and A_620._ This measurement provided an absorbance value indicating the pretreatment metabolic activity and was used to normalize the post-treatment metabolic activity. Control and lovastatin-pretreated cells were seeded into 96-well plates, treated with rituximab (10 μg/ml) in the presence of 10% human serum (as a complement source). After 1 h, Alamar blue was added to a final dilution of 1:10. The color was developed after 24 h of incubation. Cell viability was calculated as the ratio of absorbance of lovastatin-treated cells to the absorbance of control cells.

For ADCC assays, Raji cells were labeled with 100 μCi ^51^Cr (Amersham Biosciences) for 1 h. After extensive washing in PBS, the cells were adjusted to 1 × 10^5^ cells/ml and added to round-bottom microtiter plates (Greiner Bio-One). Then, rituximab (0.0005 to 2.5 μg/ml) or a control medium were added for 15 minutes followed by addition of peripheral blood mononuclear cells used as effector cells at an effector-to-target (E:T) cell ratio of 100:1. After incubation (4 h, 37 °C), assays were stopped by centrifugation, and ^51^Cr release from triplicate samples was measured as counts per minute (cpm) in a scintillation counter. Percentage of cellular cytotoxicity was calculated using the formula: % specific lysis = (experimental cpm − basal cpm) / (maximal cpm − basal cpm) × 100, where maximal ^51^Cr release was determined by adding Triton X-100 (1.5% final concentration) to target cells, and basal release was measured in the absence of sensitizing antibodies and effector cells.

### FACS Analysis

Cells at a density of 1 × 10^6^/ml were incubated with saturating amounts of FITC-conjugated mAb against CD20 (clone B9E9 [HRC20]; Beckman Coulter), and HLA-DR (clone Immu357; Beckman Coulter ), CD45 RA (clone HI100; Becton Dickinson) or PE-conjugated mAb against CD19 (clone J4.119; Beckman Coulter) and CD22 (clone SJ10.1H11; Beckman Coulter) and FITC- or PE-conjugated IgG1 (isotypic control, Beckman Coulter; 1:5 dilution) for 30 min at room temperature in the dark. Prior to analysis cells were fixed for 20 min in OptilLyse C Lysing Solution (Beckman Coulter), washed twice with PBS, and resuspended in PBS at the density 1 × 10^5^. Cells were analyzed on an EPICS/XL-MCL flow cytometer using System II software version 3.0. For rituximab binding, Raji, Ramos, or Daudi cells were incubated at room temperature for 30 min. After washing in PBS (three times), cells were resuspended in 100 μl PBS and stained in the dark for 30 min with polyclonal FITC-conjugated antibody (DAKOCytomation).

Freshly isolated leukocytes of patients with hypercholesterolemia were stained with FITC-conjugated CD20 mAb (ofatumumab, or B1) and PE-conjugated CD21 mAb (B-ly4; BD Pharmingen). Cells were incubated for 30 min at room temperature in the dark. After washing with FACS buffer, cells were analyzed on a FACS Calibur (Becton Dickinson) using Cell Quest Pro software version 5.2. CD20 mAb binding intensity was identified by gating on viable cells in the lymphogate (FSC versus SSC) and CD21^+^ cells. The mean fluorescence intensity (MFI) serves as a measure for mAb binding on a per-cell basis. The ratio of MFI of CD20/CD21 mAb staining was calculated and served as a measure for CD20 expression.

### Western Blotting

Control cells or cells incubated with 10 μM lovastatin for 48 h were washed twice with PBS, pelleted, and lysed with radioimmunoprecipitation assay buffer containing Tris base 50 mM, NaCl 150 mM, NP-40 1%, sodium deoxycholate 0.25%, and EDTA 1 mM supplemented with Complete protease inhibitor cocktail tablets (Roche Diagnostics). Protein concentration was measured using Bio-Rad Protein Assay. Equal amounts of whole-cell proteins were separated on 12.5% SDS-polyacrylamide gel, transferred onto Protran nitrocellulose membranes (Schleicher and Schuell BioScience), blocked with TBST (Tris-buffered saline [pH 7.4] and 0.05% Tween 20) supplemented with 5% nonfat milk and 5% FBS. The following mAbs (at 1:1000 dilution) were used for the overnight incubation: anti-CD20 (NCL-CD20-L26; Novocastra Laboratories), and anti–ICAM-1 (Santa Cruz Biotechnology). After extensive washing with TBST, the membranes were incubated for 45 min with peroxidase-conjugated ImmunoPure Goat Anti-Mouse IgG [F(ab′)_2_] (Jackson ImmunoResearch Laboratories). The chemiluminescence reaction for horseradish peroxidase was developed using the SuperSignal WestPico Trail Kit (Pierce) on a standard x-ray film. The blots were stripped in 0.1 M glycine (pH 2.6) and reprobed with anti-tubulin mouse mAb (Calbiochem).

### RT-PCR

Cells treated for indicated times with 10 μM lovastatin were washed twice with PBS, pelleted, and treated with 1 ml TRIzol Reagent (Invitrogen) to extract total RNA according to the manufacturer protocol. RNA concentration was measured with an Eppendorf Biophotometer. The first-strand cDNA synthesis containing 1 μg total RNA was primed with oligo(dT) using Omniscript RT Kit (Qiagen). Primers used for CD20 PCR amplification were forward: 5′ TGAATGGGCTCTTCCACATTGCC3′ and reverse: 5′ CCTGGAAGAAGGCAAAGATCAGC3′. The cycling conditions in the Mastercycler personal (Eppendorf) consisted of a first step of 94 °C denaturation for 10 min, followed by 35 cycles of annealing at 54 °C for 60 s, extension at 75 °C for 90 s, and denaturation at 94 °C for 30 s, with a final elongation step at 75 °C for 10 min using HotStar Taq DNA Polymerase (Qiagen). Amplification products were analyzed by 1.5% agarose gel electrophoresis.

### Transient siRNA Transfection

At 24 h before transfection, Raji cells were seeded from single-cell suspension at 2 × 10^5^ cells/well in a 24-well plate. After overnight culture, cells were transfected with siRNA against HMG-CoAR (sequences provided by Qiagen) using HiPerFect Transfection Reagent (Qiagen) according to the manufacturer's protocol.

### Immunofluorescence

Control and lovastatin-pretreated cells were stained in suspension at a density of 5 × 10^5^/ml with anti-CD20 FITC-conjugated mAb (1:10 in PBS; Immunotech Coulter) for 30 min at room temperature. After washing in PBS (three times), 200 μl of the cell suspension was spun onto a cytospin slide. The slides were air-dried, acetone-fixed for 15 min at room temperature, washed 3 times with PBS, and incubated with anti-CD20 mAb (Novocastra; 1:100 in PBS with 5% normal donkey serum [Jackson]) for 60 min at room temperature. The slides were washed three times in PBS and incubated with donkey anti-mouse Alexa555-conjugated antibody (Molecular Probes; 1:200 for 30 min at room temperature). The slides were washed, mounted in Vectashield (Vector Laboratories), and examined by fluorescence microscopy (Leica TCS SP2).

### Surface Protein Biotinylation

Control and lovastatin-treated cells, washed three times with ice-cold PBS (pH 8.0) and resuspended at a density of 25 × 10^6^ cells/ml, were surface-labeled with 2 mM (final concentration) EZ-link sulfo-NHS-biotin (Pierce) for 30 min at room temperature. Cells were washed three times (in PBS with 100 mM glycine) and lysed with radioimmunoprecipitation assay lysis buffer containing protease inhibitors (as described earlier). Next, samples were mixed for 1 h at room temperature with immobilized NeutrAvidin Protein (Pierce) to separate the biotinylated surface protein from non-biotinylated surface protein. After five washings, gel-bound complexes were boiled in 2× Laemmli sample buffer and analyzed for CD20 by Western blotting using anti-CD20 mAb (Novocastra) as described above.

### Atomic Force Microscopy

The measurements were performed using a custom-made atomic force microscope described in more detail elsewhere [26]. The device used for the experiment was equipped with a “liquid cell” setup to assure native conditions. Commercially available silicon nitride cantilevers with the spring constant of 0.01 N/m (MLCT-AUHW; Veeco Probes) and a nominal tip radius of about 20 nm were used as probes. All measurements were performed in PBS buffer at room temperature. Two types of experiments were performed: (i) control measurements where the antigen-antibody interaction was studied between rituximab attached to the atomic force microscopy (AFM) probe and CD20 present on a surface of intact Raji cells; and (ii) measurements with rituximab attached to the AFM probe and CD20 present on a surface of Raji cells, previously incubated for 48 h with 10 μM lovastatin. Force curves, reflecting the relationships between the cantilever deflection and the relative sample position, were recorded at the constant retraction velocity of 4.6 μm/s. The determined system spring constant was about 0.00032 N/m. Thus, the loading rate defined as a product of these two values resulted in 1,472 piconewton (pN)/s. The total number of the curves was about 1,500 for each measurement type. The measurements were repeated three times with newly immobilized antibody and a new sample (Raji cells attached to the poly-L-lysine–coated glass coverslips). In order to assure that the force measured by AFM originated from the specific interaction between the antibody (rituximab) and its antigen CD20 present on the cell plasma membrane, the antigens were blocked during the course of 1-h incubation of lovastatin-treated cells with PBS buffer containing 10 μg/ml of the antibody. Next, cells were washed and immediately measured with AFM using the antibody functionalized AFM cantilever. The functionalization protocol was described earlier [27]. Briefly, standard silicon nitride cantilevers were cleaned in acetone for 15 minutes and then irradiated with UV light for 30 minutes. After overnight silanization with 3-aminopropyltriethoxysilane (APTES; Sigma Aldrich) in a desiccator, cantilevers were immersed in glutaraldehyde (Fluka) dissolved in PBS (2.5%) for 30 minutes. Afterwards, they were washed with PBS buffer and functionalized by sinking in the antibody solution (10 μg/ml) for 1 h.

### Digestion of Surface Proteins with Trypsin and Chymotrypsin

Control and lovastatin-treated Raji cells (2 × 10^6^) were rinsed with PBS, centrifuged under mild conditions (60*g* for 5 min), and suspended in 100 μl PBS (pH 7.4). Cells were incubated for 10 min at 37 °C with 0, 0.5, 1.5, 2, and 2.5 μg of bovine pancreas trypsin (T; Sigma Aldrich) or chymotrypsin (ChT; Sigma Aldrich). After enzyme inactivation with 20 μg soybean trypsin inhibitor (Sigma Aldrich), cells were centrifuged (60*g* for 5 min), lysed by boiling in Laemmli sample buffer, and subjected to SDS-PAGE. Separated proteins were transferred to a nitrocellulose membrane and probed with anti-CD20 (Novocastra) and anti-tubulin (Calbiochem) mAbs.

### Statistical Analysis

Data from cytotoxicity experiments were calculated using Microsoft Excel 98 and represent mean ± standard deviation. Differences in cytotoxicity assays were analyzed for significance by Student's *t* test. Significance was defined as a two-sided *p* < 0.05. All experiments using cell lines were performed at least three independent times, the AFM experiment was performed twice, and the experiments using freshly isolated (patient) cell material were performed once but always in duplicate.

## Results

### Statins Abrogate Antitumor Activity of Rituximab Against Raji B Cell Lymphoma Cells

**Figure 1:**
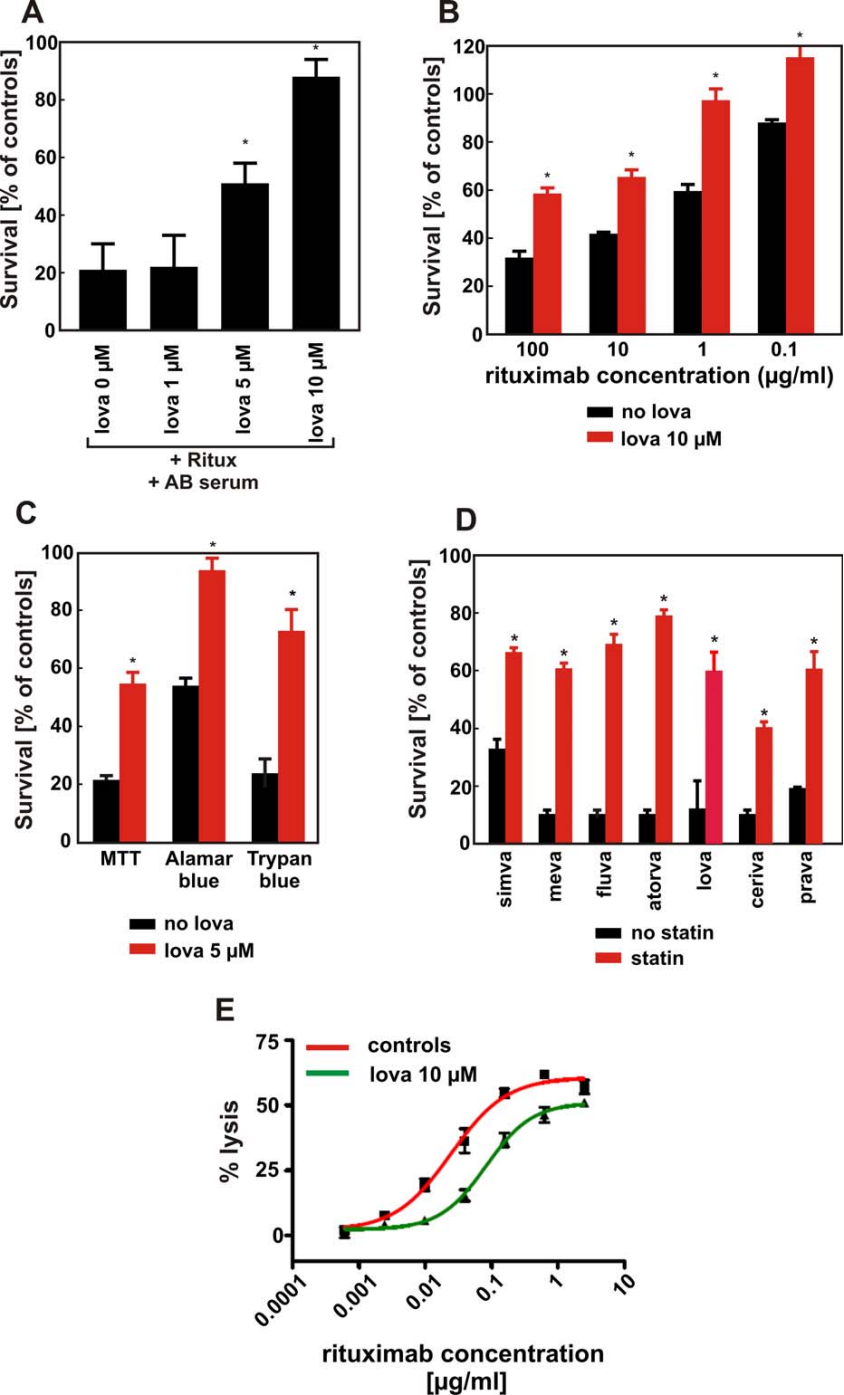
Statins Protect Raji Cells Against Rituximab-Mediated CDC (A) Raji cells were incubated with either diluent or increasing doses of lovastatin (from 1 to 10 μM) for 48 h. Then, equal numbers of cells (1 × 10^5^/well) were incubated for 60 min with 10 μg/ml rituximab in the presence of 10% human AB serum as a complement source. Cell viability was measured in a MTT assay. The survival of cells is presented as percentage of corresponding diluent- or statin-pretreated cells without rituximab. (B) Raji cells were incubated with either diluent or lovastatin at 10 μM concentration for 48 h. Then, equal numbers of cells (1 × 10^5^/well) were incubated for 60 min with serial dilutions (from 100 to 0.1 μg/ml) of rituximab in the presence of 10% human AB serum a complement source. Cell viability was measured in a MTT assay. The survival of cells is presented as percentage of corresponding diluent- or statin-pretreated cells without rituximab. (C) Raji cells were incubated with either diluent or 5 μM lovastatin for 48 h. Then, equal numbers of cells (1 × 10^5^/well) were incubated for 60 min with 10 μg/ml rituximab in the presence of 10% human AB serum as a complement source. Cell viability was measured with MTT assay, Alamar blue assay, and trypan blue exclusion assay. (D) Raji cells were incubated with either diluent or simvastatin (10 μM), mevastatin (10 μM), fluvastatin (10 μM), atorvastatin (10 μM), lovastatin (10 μM), cerivastatin (1 μM), or pravastatin (10 μM) for 48 h. Then, equal numbers of cells (1 × 10^5^/well) were incubated for 60 min with 10 μg/ml rituximab in the presence of 10% human AB serum as a complement source. Cell viability was measured with MTT assay. (E) Raji cells were incubated with either diluent or lovastatin at 10 μM concentration for 48 h. Then, after labeling with ^51^Cr, equal numbers of target cells (1 × 10^5^/ml) were incubated with serial dilutions of rituximab (from 2.5 to 0.0005 μg/ml) and effector peripheral blood mononuclear cells at E:T = 100:1 for 4 h. *p < 0.001 (two-way Student's *t*-test) as compared with controls.

Complement-dependent cytotoxicity (CDC) contributes significantly to the clinical efficacy of rituximab. Therefore, the potential of rituximab to induce CDC of Raji cells was assessed in a MTT assay after 1-h incubation in the presence of human AB serum as a complement source. Incubation of Raji cells with rituximab (10 μg/mL) and 10% human AB serum resulted in killing of 60%–80% of lymphoma cells. Neither rituximab nor AB serum alone affected viability of tumor cells under these experimental conditions (not shown). A 48-h preincubation of Raji cells with lovastatin resulted in a dose-dependent reduction of rituximab-mediated CDC (Figure 1A). As a control, lovastatin treatment alone did not affect the viability of Raji cells (unpublished data). The protection of Raji cells against CDC following preincubation with lovastatin was observed over a dose range of rituximab concentrations (0.1–100 μg/ml; Figure 1B). The reduced cell lysis was confirmed when viability of lymphoma cells preincubated with 5 μM lovastatin was examined by other methods, including a trypan blue exclusion assay or a fluorescent Alamar blue assay (Figure 1C). Because these results were consistent, we used the MTT assay for evaluating cell viability in further experiments.

Statins are structurally related, but they have several unique pharmacodynamic properties that result in pleiotropic, presumably HMG-CoAR–independent effects [28]. Therefore, we examined the influence of other statins on rituximab-mediated CDC of Raji cells. A 48-h incubation of Raji cells with all additionally investigated statins (atorvastatin, cerivastatin, fluvastatin, mevastatin, pravastatin, and simvastatin) conferred resistance to rituximab-mediated CDC (Figure 1D).

ADCC is another important mechanism involved in rituximab-mediated cytotoxicity. Control and lovastatin-pretreated ^51^Cr-labelled lymphoma cells were used as targets in an ADCC reaction with peripheral blood mononuclear cells as effectors. A 48-h incubation of Raji cells with 10 μM lovastatin impaired ADCC across a range of rituximab concentrations (0.0005–2.5 μg/ml; Figure 1E).

### Lovastatin Does Not Change the Expression Level of CD20

**Figure 2:**
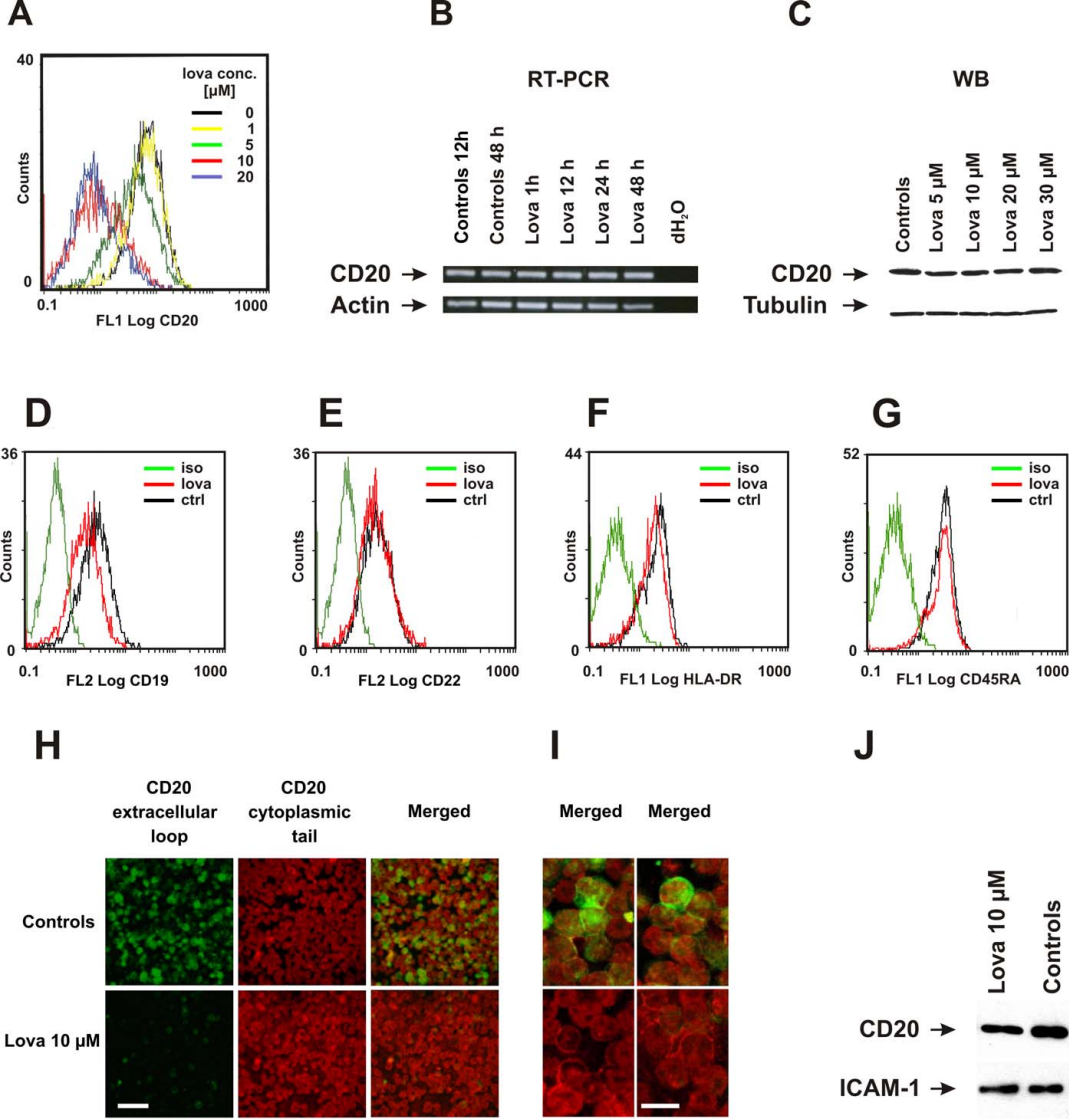
The Influence of Lovastatin on the Expression of CD20 in Raji Cells (A) Raji cells were incubated with either diluent (line indicating 0 μM) or lovastatin (1–20 μM) for 48 h. Then, 1 × 10^6^/ml of cells were incubated with saturating amounts of FITC-conjugated anti-CD20 mAb (B9E9) or IgG1 (isotype control) for 30 min at room temperature in the dark. Binding of mAb to Raji cells was measured with flow cytometry. (B) CD20 mRNA levels in Raji cells incubated with either diluent (controls) or 10 μM lovastatin for 1 to 48 h assayed by RT-PCR. (C) CD20 protein levels in Raji cells incubated with either diluent or 5, 10, 20, and 30 μM lovastatin for 48 h and assayed by Western blotting (NCL-CD20-L26 mAb). (D–G) Raji cells were incubated with either diluent (ctrl) or 10 μM lovastatin for 48 h. Then, 1 × 10^6^/ml of cells were incubated with saturating amounts of PE-conjugated anti-CD19 mAb (D), PE-conjugated anti-CD22 mAb (E), FITC-conjugated anti-HLA-DR mAb (F), FITC-conjugated anti-CD45RA mAb (G), or IgG1 (isotypic control) for 30 min at room temperature in the dark. Binding of mAb to Raji cells was measured with flow cytometry. (H) Immunofluorescence studies of Raji cells incubated for 48 h with either diluent or 10 μM lovastatin. Raji cells were incubated with FITC-conjugated (green) mAb (B9E9) to detect the extracellular (conformational) epitope located in the larger CD20 loop. Upon washing with PBS, the cells were permeablized with acetone and incubated with IgG1 mAb (NCL-CD20-L26) directed against a cytoplasmic (linear) epitope within CD20 molecule. After washing, a secondary Alexa555-labeled (red) anti-IgG1 (anti–NCL-CD20-L26) mAb was used. Bar = 50 μm. (I) Higher magnification of Raji cells stained with both extracellular and intracellular epitopes (two different areas are shown for each group). Bar = 10 μm. (J) Control or lovastatin-treated (10 μM for 48 h) Raji cells (1 × 10^6^) were incubated with EZ-link sulfo-NHS-biotin. Total cellular lysates were precipitated with immobilized NeutrAvidin protein followed by electrophoresis and blotting with anti-CD20 (NCL-CD20-L26 mAb) and anti–ICAM-1 mAb.

In addition to lowering of cholesterol synthesis, statins exert pleiotropic effects, thereby influencing the expression of a number of different genes [28–30]. Therefore, we hypothesized that statin-mediated effects might result from down-regulating CD20 expression. To evaluate the influence of statins on CD20 expression, flow cytometry was performed using a FITC-conjugated mAb (clone B9E9) that binds to an extracellular conformational epitope of CD20. These studies revealed a significantly decreased binding of anti-CD20 mAb to Raji cells incubated with a range of lovastatin concentrations (from 1–20 μM). While more than 95% of control Raji cells stained positive for CD20, only 30% of cells showed significant binding to CD20 after a 48-h pretreatment with 10 μM lovastatin (Figure 2A). Simultaneously, staining for other B cell markers (CD22, HLA-DR, and CD45RA) in lovastatin-treated Raji cells did not change, and CD19 staining was only slightly decreased (Figure 2D–2G). In contrast, we did not observe reductions of CD20 mRNA levels in time course (Figure 2B) and dose-titration (unpublished data) experiments with lovastatin-treated Raji cells. Also, a 48-h incubation with different concentrations of lovastatin (from 5–30 μM) did not influence the total amount of CD20 protein in the cells, as determined by Western blotting (Figure 2C).

Decreased binding of CD20 mAb in the absence of the reduction in CD20 mRNA and protein levels might be explained by a retention/redistribution of CD20 into cytosolic compartments, its shedding from the plasma membrane or other mechanisms. To examine the subcellular localization of CD20 in Raji cells, we performed a double immunofluorescence staining with anti-CD20 mAb directed against an extracellular epitope of CD20, and, after cell permeabilization, with a mAb against a linear intracellular epitope. While in untreated controls both mAb detected CD20 expressed on Raji cells, a 48-h pretreatment with lovastatin nearly completely abrogated binding of mAb directed to the extracellular epitope (Figure 2H). However, mAb directed against the cytosolic tail of CD20 retained its membrane staining (Figure 2I). These experiments indicate that in lovastatin-treated cells, CD20 is still present in the plasma membrane.

To further elucidate these observations, we labeled control and lovastatin-treated cells with EZ-link sulfo-NHS-biotin. This water-soluble and membrane impermeable reagent stably binds to primary amino (-NH_2_) groups of extracellular portions of membrane proteins. Labeled cells were lysed and biotinylated proteins were precipitated with immobilized NeutrAvidin protein. Electrophoresis of the precipitates followed by blotting with anti-CD20 mAb (targeting the cytoplasmic portion of CD20) revealed that CD20 in lovastatin-treated cells is detectable in comparable, albeit slightly lower levels as compared with controls (Figure 2J). The expression of ICAM-1, another transmembrane protein, was identical in control and lovastatin-treated cells (Figure 2J). Therefore, these studies confirm that CD20 remains in the plasma membrane of lovastatin-treated cells.

### Impaired Rituximab-Mediated CDC in Cholesterol-Depleted Cells Is Not Dependent on Disruption of Lipid Rafts

**Figure 3:**
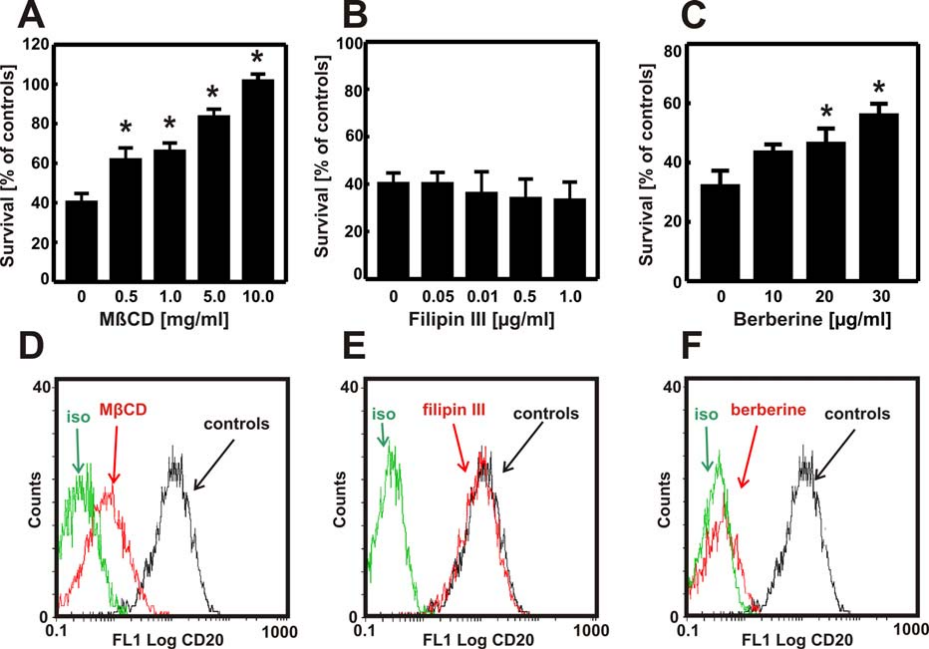
The Influence of Cholesterol-Modulating Drugs on the Expression of CD20 in Raji Cells (A–C) Raji cells were incubated with either diluents or MβCD (0–10 mg/ml) for 30 min, filipin III (0–1 μg/ml) for 30 min, or berberine (0–30 μg/ml) for 24 h. Then, equal numbers of cells (1 × 10^5^/well) were incubated for 60 min with 10 μg/ml rituximab in the presence of 10% human AB serum as a complement source. Cell viability was measured in a MTT assay. (D–F) Raji cells were incubated with either diluents or MβCD (5 mg/ml) for 30 min, filipin III (0.5 μg/ml) for 30 min, or berberine (25 μg/ml) for 24 h. Then, 1 × 10^6^/ml cells were incubated with saturating amounts of FITC-conjugated anti-CD20 mAb (B9E9) or IgG1 (isotype control) for 30 min at room temperature in the dark. Control indicates the cells not incubated with MβCD, filipin III, and berberine, respectively. *p < 0.001 (two-way Student's *t*-test) as compared with controls. Binding of mAb to Raji cells was measured with flow cytometry.

Binding of rituximab to CD20 has been shown to translocate CD20 to cholesterol-rich microdomains within the plasma membrane; this effect was associated with induction of apoptosis of lymphoma cells and activation of CDC [22,23]. As statins, by inhibiting cholesterol biosynthesis, have been shown to interfere with the formation of lipid rafts [11], we hypothesized that the impaired rituximab-mediated CDC might result from the inability to translocate CD20 to these microdomains. To verify this hypothesis, Raji cells were incubated with MβCD and filipin III, which disrupt lipid rafts via different mechanisms: MβCD extracts cholesterol from the plasma membrane, whereas filipin III binds stoichiometrically to cholesterol, thereby introducing additional charge that forces cholesterol molecules to distribute evenly within the plasma membrane [31]. These two reagents produced completely different effects in our studies. While MβCD abrogated rituximab-mediated CDC (Figure 3A), filipin III had no effect (Figure 3B). Interestingly, berberine—an agent that inhibits cholesterol synthesis but in an HMG-CoAR–independent mechanism [32]—significantly decreased rituximab-mediated CDC (Figure 3C). Accordingly, we have also observed that a 30-min incubation with MβCD (Figure 3D) and a 24-h incubation with berberine (Figure 3F) but not filipin III (Figure 3E) impaired binding of anti-CD20 mAb to Raji cells. These results indicate that the presence of plasma membrane cholesterol and not lipid rafts are critical for binding of anti-CD20 mAb to lymphoma cells as well as for rituximab-mediated CDC of lymphoma cells.

### Impaired Binding of Anti-CD20 mAb Results from Inhibition of HMG-CoAR and Is Observed for Type I and Type II Anti-CD20 mAb

**Figure 4:**
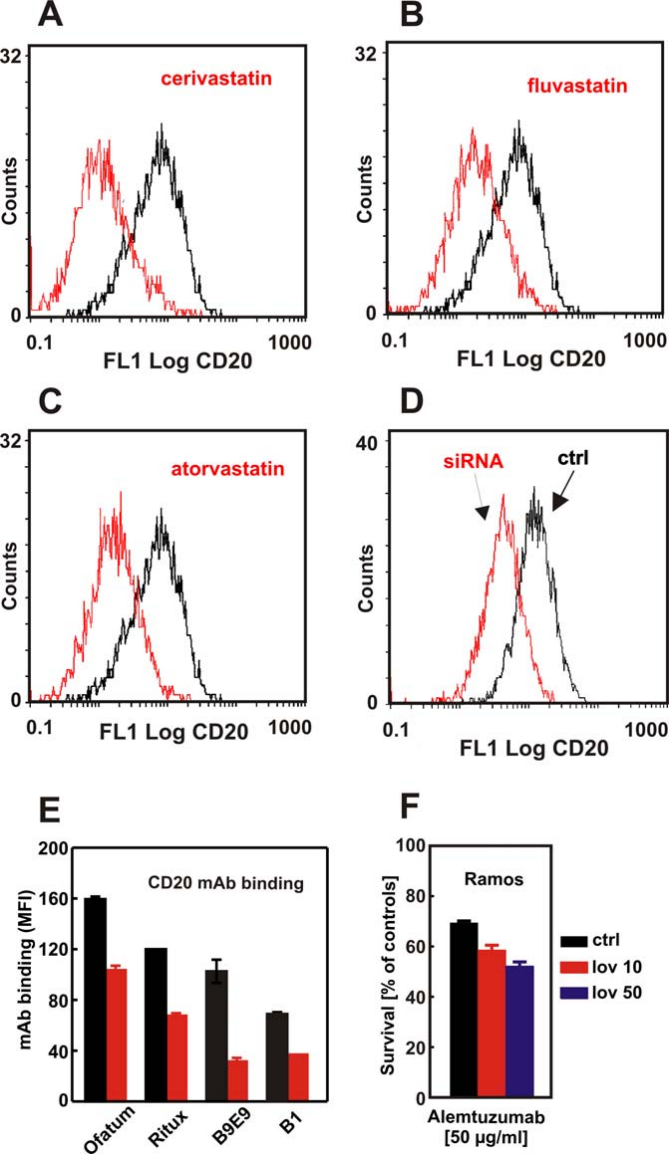
The Influence of Statins, FTI, and GGTI Inhibitors and siRNA on Antibody-Mediated CDC and/or CD20 Detection Raji cells were incubated with either diluent or 1 μM cerivastatin (A), 10 μM fluvastatin (B), or 10 μM atorvastatin (C) for 48 h. Then, 1 × 10^6^/ml of cells were incubated with saturating amounts of FITC-conjugated anti-CD20 mAb (B9E9) or IgG1 (isotypic control) for 30 min at room temperature in the dark. Binding of mAb to Raji cells was measured with flow cytometry. (D) Raji cells were transfected with siRNA targeting HMG-CoAR or irrelevant siRNA using OligofectAMINE. After 48 h, 1 × 10^6^/ml of cells were labeled as described above. (E) Daudi cells were incubated with either diluent or 10 mg/ml MβCD for 30 min. Then, 1 × 10^5^/ml of cells were incubated with saturating amounts of the FITC-conjugated anti-CD20 mAbs ofatumumab, rituximab, B1, and B9E9 for 30 min at room temperature in the dark. The MFI corrected for the FITC–protein ratio was measured with flow cytometry. (F) Ramos cells were incubated with either diluent or lovastatin (10 and 50 μM) for 48 h. Then, equal numbers of cells (1 × 10^5^/well) were incubated for 60 min with 10 μg/ml alemtuzumab in the presence of 10% human AB serum as a complement source. Cell viability was measured in a MTT assay. The survival of cells is presented as percentage of corresponding diluent- or statin-pretreated cells without alemtuzumab.

In subsequent studies, we investigated the influence of other statins on binding of anti-CD20 mAb to Raji cells. Incubation of Raji cells with cerivastatin (Figure 4A), fluvastatin (Figure 4B), and atorvastatin (Figure 4C), as well as simvastatin and mevastatin (unpublished data), resulted in analogous effects to lovastatin, indicating that detection of CD20 and anti-CD20–mediated effector functions are strongly associated with inhibition of HMG-CoAR. To further exclude a possibility that the presence of drugs might interfere with antibody binding, the expression of HMG-CoAR was silenced with siRNA. Inhibition of HMG-CoAR expression resulted in a decreased binding of anti-CD20 mAb to Raji cells (Figure 4D).

Downstream products of HMG-CoAR activity are isoprenoid compounds that include farnesyl and geranylgeranyl pyrophosphates used for posttranslational protein modification. A 48-h preincubation of Raji cells with inhibitors of FTI and GGTI did not decrease CD20-mediated CDC (unpublished data), indicating that the effects of statins are independent from impaired prenylation of intracellular proteins. As a control, FTI and GGTI alone only modestly affected the viability of Raji cells.

To study whether our observations were restricted to rituximab and B9E9, we also tested ofatumumab (a type I CD20 mAb directed to a distinct CD20 epitope) and the type II CD20 mAb B1 [33] for CD20 binding and CDC after cholesterol depletion. Indeed, MβCD treatment also decreased CD20 binding of both ofatumumab and B1 to Daudi cells (Figure 4E) and Raji cells (ofatumumab and rituximab, ∼50% reduction; B1 and B9E9, ∼80% reduction). However, MβCD treatment affected ofatumumab-mediated CDC less severely compared with rituximab, especially for Raji cells (with 60% versus 82% of cells surviving respectively; unpublished data).

Importantly, preincubation of Ramos cells with 10 or 50 μM lovastatin for 48 h did not impair alemtuzumab-mediated CDC (Figure 4F). Alemtuzumab is a mAb approved for clinical use that binds CD52, which is a GPI-anchored glycoprotein expressed on the surface of lymphoma cells.

**Figure 5:**
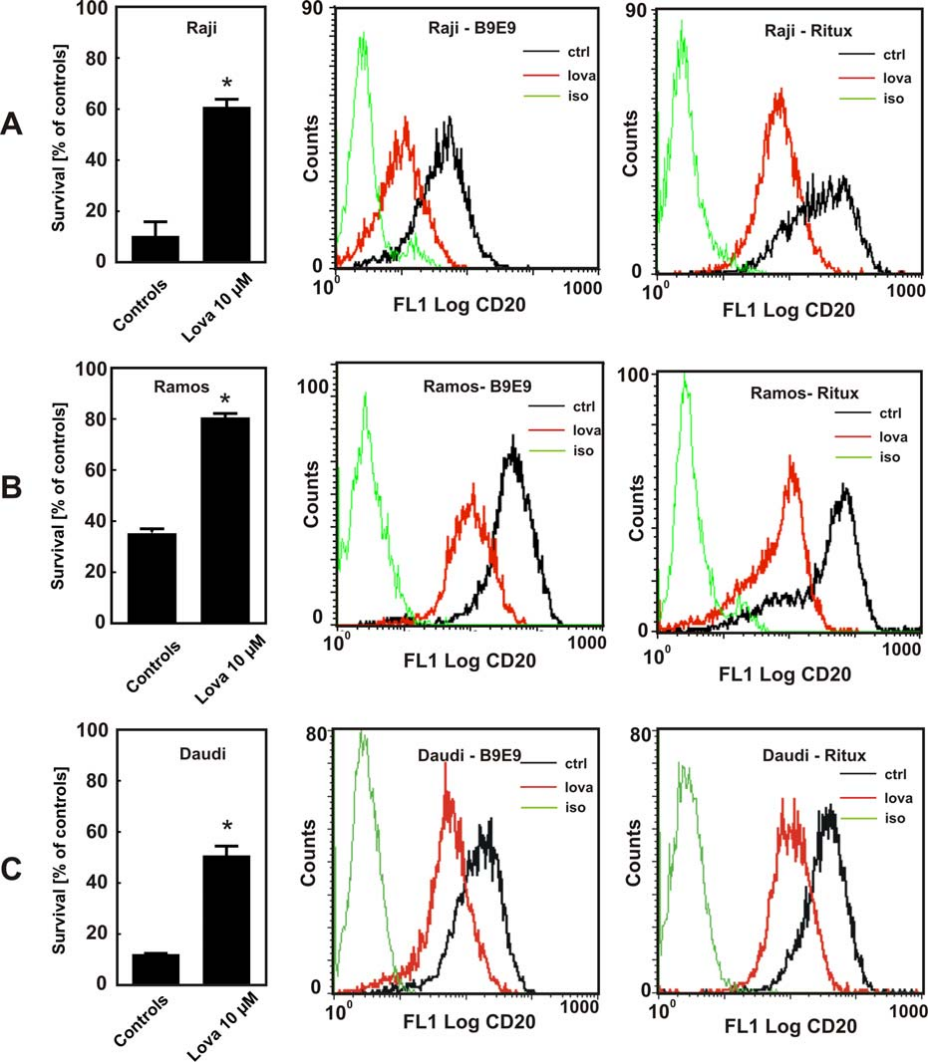
Correlation Between Reduced Anti-CD20 mAb Binding and Decreased Rituximab-Mediated CDC in Lovastatin-Treated Cells Raji (A), Ramos (B), or Daudi (C) cells were incubated with either diluent or lovastatin (10 μM) for 48 h. Then, equal numbers of cells (1 × 10^5^/well) were incubated for 60 min with 10 μg/ml rituximab in the presence of 10% human AB serum as a complement source. Cell viability was measured in a MTT assay. The survival of cells is presented as percentage of corresponding diluent- or statin-pretreated cells without rituximab (left graphs). Raji, Ramos, and Daudi cells were incubated with either diluent or lovastatin (10 μM) for 48 h. Then, 1 × 10^6^/ml of cells were labeled with FITC-conjugated B9E9 (center graphs) or rituximab (right graphs) and a secondary FITC-conjugated anti-IgG1 antibody. *p < 0.001 (two-way Student's *t*-test) as compared with controls.

Studies with two additional lymphoma cell lines, Ramos and Daudi, showed a similar decrease in rituximab and B9E9 binding as compared with that observed with Raji cells (Figure 5A–5C). The decrease in rituximab binding upon lovastatin treatment correlated with a decreased efficacy of CDC (Figure 5A–5C).

### Cholesterol Is Necessary for CD20 Binding and Rituximab-Mediated CDC

**Figure 6:**
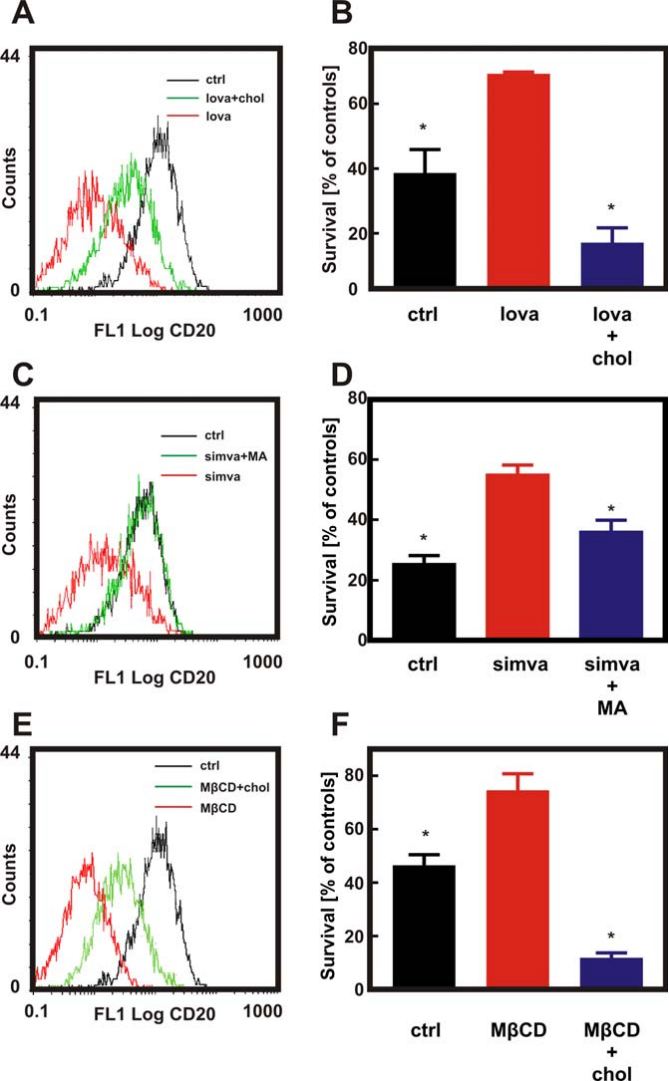
Cholesterol Is Necessary for CD20 Binding and Rituximab-Mediated CDC Raji cells were incubated with either diluent or lovastatin (10 μM for 48 h [A and B]), simvastatin (10 μM for 48 h [C and D]) or MβCD (5 mg/ml for 30 min [E and F]). Water-soluble cholesterol (chol; 0.2 mg/ml) was added for 2 h prior to flow cytometry analysis (A, E) or addition of 10 μg/ml rituximab and 10% human AB serum (B and F). Mevalonic acid (MA) was added together with simvastatin at a 200 μM concentration (C and D). Then for flow cytometry studies (A, C, and E), 1 × 10^6^/ml of cells were incubated with saturating amounts of FITC-conjugated anti-CD20 mAb (B9E9) or IgG1 (isotype control) for 30 min at room temperature in the dark. Cell viability was measured in a MTT assay (B, D, and F). The survival of cells is presented as percentage of corresponding diluent- or statin-pretreated cells without rituximab. *p < 0.001 (two-way Student's *t*-test) as compared to statin-treated (B and D) or MβCD-treated (F) cells.

To further investigate the role of cholesterol in binding of anti-CD20 mAb to the target antigen and in rituximab-mediated CDC, we performed restitution experiments. Preincubation of Raji cells with 10 μM lovastatin followed by a 30-min incubation with exogenous cholesterol significantly restored or improved binding of anti-CD20 mAb (Figure 6A). Similarly, a 30-min incubation of Raji cells with cholesterol after a 48-h pretreatment with 10 μM lovastatin completely restored sensitivity of lymphoma cells to rituximab-mediated CDC (Figure 6B). Accordingly, co-treatment of Raji cells with mevalonic acid, a direct product of HMG-CoAR, completely restored binding of anti-CD20 mAb (Figure 6C) as well as rituximab-mediated CDC of statin-treated cells (Figure 5D). Similar results were obtained with exogenous cholesterol that significantly reversed MβCD-induced effects (Figure 6E and 6F).

### Lovastatin Changes Conformation of CD20

Impaired rituximab-mediated CDC as well as impaired binding of anti-CD20 mAb to Raji cells incubated with 10 μM lovastatin for 48 h did not seem to result from decreased expression or shedding of CD20 from plasma membrane. Rapid (within 30 min) restoration of rituximab-mediated CDC after the addition of cholesterol indicates that CD20 might undergo cholesterol-dependent conformational changes. Association of CD20 with the plasma membrane prevents the use of direct crystallography to study conformational changes of this molecule. To address this issue, we therefore performed studies with AFM. Changes of CD20 conformation were furthermore studied by performing limited proteolysis.

**Figure 7:**
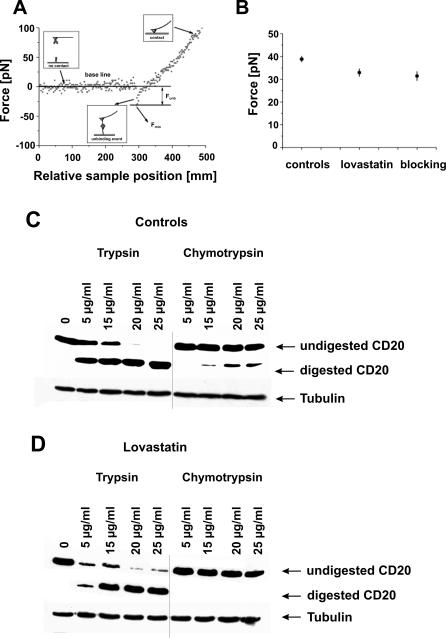
Lovastatin Changes Conformation of CD20 in Raji Cells (A) The force curve obtained for the interaction between rituximab attached to the AFM tip and the antigen (CD20) present on the surface of living cells is shown. The unbinding force *F_unb_* was determined as a difference between the base line and the force value *F_min_* at the moment of bond rupture. (B) The unbinding force calculated for the interaction between the mAb-coated AFM tip and the antigen (CD20) on cell surface was assessed in a control measurement, lovastatin treatment, and after blocking of the antigen binding sites by soluble rituximab, respectively. The estimated error is a standard deviation of the mean (SEM). Control (C) and lovastatin-pretreated (D) Raji cells were treated with increasing concentrations of trypsin and chymotrypsin. Cell lysates were separated in SDS-PAGE and probed with anti-CD20 (NCL-CD20-L26 mAb) detecting the intracellular part of CD20 antigen.

For AFM measurements, rituximab was attached to an AFM probe, and Raji cells were immobilized onto coverslips. The binding force of immobilized rituximab to CD20 was assessed using Raji cells preincubated for 48 h with 10 μM lovastatin. As a control, the experiment was repeated with diluent-treated Raji cells and lovastatin-treated cells on which the available binding sites were blocked with soluble rituximab (at 10 μg/ml). The unbinding force of a single pair of molecules can be directly measured by AFM with pN resolution. Its value is characteristic for the studied interaction under the given experimental conditions. A stronger unbinding force indicates a stronger molecular interaction and thereby indicates a higher affinity binding of rituximab to the surface of Raji cells. A typical force curve of an unbinding event is presented in Figure 7A. Its main characteristics were similar before and after treatment with lovastatin. A common method of determining the unbinding force for a molecular interaction is to attribute the position of the first peak in a histogram obtained to the unbinding event of a single molecular complex [34]. Here, it is assumed that the unbinding probability is lower than 30% and that only a few molecular bonds are expected to have formed. Figure 7B presents the unbinding force calculated on the basis of these force histograms. The unbinding force calculated for a single antibody–antigen complex determined in the control experiment was larger (38.8 ± 1.3 pN) than after lovastatin treatment (32.9 ± 1.7 pN) and after blocking available binding sites with soluble rituximab (31.4 ± 2.0 pN).

Limited proteolysis of the surface proteins by T and ChT is another indirect method for determining conformational changes [35]. Control and lovastatin-pretreated Raji cells were incubated with increasing concentrations of T and ChT. Total cellular lysates were then separated in SDS-PAGE and probed with anti-CD20 mAb detecting an intracellular part of CD20 antigen. In control cells, incubations with 15 μg/ml ChT resulted in digestion of CD20 protein (a lower band appears in Figure 7C); at higher concentrations, the intensity of the lower band increases. In contrast, in lovastatin-pretreated cells, CD20 is not digested even by the highest ChT concentration (Figure 7D). The susceptibility to T digestion of control and lovastatin-pretreated cells seems to be similar, although there are some important differences. Similarly to ChT digestion, lovastatin-pretreated cells seem to be less susceptible to T treatment. The upper band (representing undigested protein) disappears in samples of control cells treated with 20 and 25 μg/ml of the enzyme, while it is still present in samples of lovastatin-pretreated cells. We excluded the possibility that lovastatin inhibits ChT and T activity by performing the limited proteolysis of control cells in the presence of both proteases and 10 μM lovastatin (unpublished data). Altogether, it seems that the observed cholesterol-dependent effects are due to conformational changes in CD20 antigen, affecting the binding of type I and type II anti-CD20 antibodies.

### Cholesterol Depletion Impairs Anti-CD20 Binding and Rituximab-Mediated CDC of Freshly Isolated Human B Cell Lymphoma Cells

**Figure 8:**
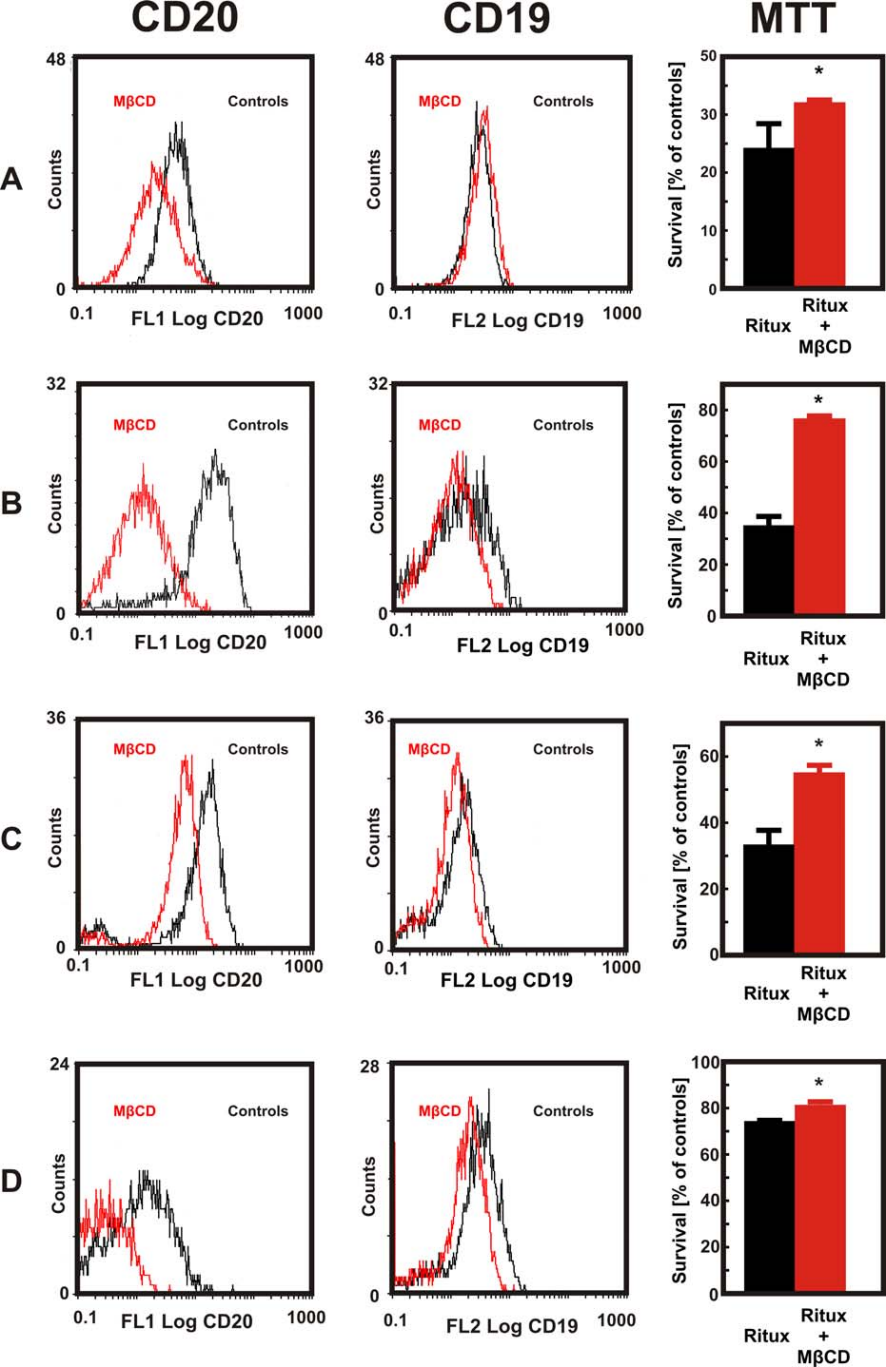
The Influence of MβCD on the Expression of CD19 and CD20 As Well As on Rituximab-Mediated CDC Against Primary Human Lymphoma Cells Primary human CD20^+^ B cell lymphoma cells—mantle cell lymphomas in (A–C), and small B cell lymphoma in (D)—were incubated with either diluent or MβCD (10 mg/ml) for 30 min. Then, 1 × 10^6^ cells/ml were incubated with saturating amounts of FITC-conjugated anti-CD20 mAb (B9E9, left graphs), PE-conjugated anti-CD19 mAb (center graphs), or IgG1 (isotype control) for 30 min at room temperature in the dark. For the rituximab-mediated CDC, human CD20^+^ B cell lymphoma cells were incubated with either diluent or 10 mg/ml MβCD for 30 min. Then, equal numbers of cells (1 × 10^5^/well) were incubated for 60 min with 400 μg/ml rituximab in the presence of 10% human AB serum as a complement source. Cell viability was measured in a MTT assay. The survival of cells is presented as percentage of corresponding diluent- or MβCD-pretreated cells without rituximab (right graphs). *p = 0.032 for (A), *p* < 0.001 for (B) and (C), and *p* = 0.041 for (D) (two-way Student's *t*-test) as compared with controls.

To further investigate the effect of cholesterol depletion on immunotherapy, we studied whether MβCD treatment reduced the capacity of rituximab to lyse freshly isolated cells of patients with B cell lymphoma. In all four patients with B cell lymphoma studied (three with mantle cell lymphoma and one with small B cell lymphoma), we observed that a 30-min incubation of lymphoma cells with 10 mg/ml MβCD significantly decreased binding of anti-CD20 (B9E9) mAb, while binding of anti-CD19 mAb was affected to a lesser extent (Figure 8). Accordingly, incubation with MβCD significantly decreased rituximab-mediated CDC of B cell lymphoma cells (Figure 8).

### Short-Term Statin Treatment Reduces Binding of Anti-CD20 mAb to the Surface of Peripheral Blood B Cells in Patients

**Figure 9:**
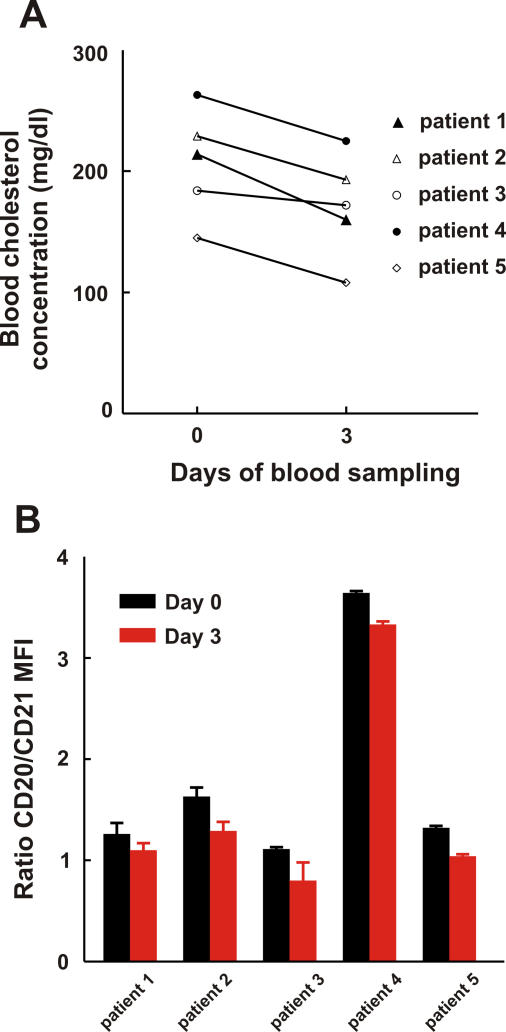
Short-Term Atorvastatin Treatment Reduces Binding of Anti-CD20 mAb to the Surface of Peripheral Blood B Cells in Patients Five patients were treated with 80 mg atorvastatin daily for three consecutive days. (A) Changes in blood cholesterol concentration before and after atorvastatin treatment of individual patients (indicated with different symbols). (B) Freshly isolated B cells of hypercholesterolemic patients were stained with FITC-conjugated anti-CD20 mAb (ofatumumab) and PE-conjugated anti-CD21 mAb (clone B-Ly4) on day 0 and day 3. CD20 mAb binding intensity (MFI) was correlated to CD21 mAb binding intensity by calculating the ratio of CD20/CD21 MFI.

To extend our observations, we studied the influence of cholesterol depletion on freshly isolated B cells from patients originally diagnosed with hypercholesterolemia who were treated with atorvastatin. A small exploratory clinical study was performed in which five patients were treated with a daily dose of 80 mg atorvastatin in order to reduce their cholesterol blood level and to determine the effect on anti-CD20 mAb binding on freshly isolated B cells using flow cytometry with anti-CD20 mAbs and a PE-conjugated anti-CD21 mAb. CD21 is an antigen expressed on B cells that was unaffected by cholesterol depletion on both B cell lines (Daudi and Raji) and freshly isolated B cell–enriched cell populations of three independent donors (unpublished data). Therefore, anti-CD21 mAb binding was considered to be an indicator for the amount of B cells present per staining. The ratio of MFI of CD20/CD21 mAb staining was calculated and served as a relative measure for CD20 expression. Figure 9A shows that atorvastatin treatment led to a significant reduction in blood cholesterol levels in all five patients (the average reduction in blood cholesterol level was 17.5% ± 3.6%; *p* < 0.01, Student's *t*-test). Freshly isolated B cells of patients stained with FITC-conjugated anti-CD20 mAb (ofatumumab) showed a decrease of 18.2% ± 3.5% (*p* = 0.01, Student's *t*-test) in binding for all five patients when compared with day 0 (pretreatment; Figure 9B). Similarly, staining with FITC-conjugated anti-CD20 mAb B1 resulted in a decreased CD20/CD21 MFI ratio for three out of five patients (unpublished data). Our results suggest that short-term statin usage lowers anti-CD20 mAb binding to B cells.

## Discussion

We observed that statins can indirectly affect conformation of CD20 molecules expressed on the surface of normal and malignant B lymphocytes. Conformational changes in CD20 molecules impair their recognition by mAbs, thereby compromising rituximab-mediated CDC and ADCC against lymphoma cells. CD20 is an integral membrane protein with tetraspanning topology, in which both the C and N termini are located within the cell [36]. It is predicted to have two extracellular loops: a very small loop between the first and second transmembrane regions, and a larger loop of approximately 44 extracellular amino acids located between the third and fourth transmembrane regions [36]. Most anti-CD20 mAbs, including the rituximab, B1, and B9E9 studied in this work, recognize overlapping epitopes within the larger loop [33]. Mutagenesis studies revealed that the region surrounding alanine 170 (A170) and proline 172 (P172) is essential for rituximab binding [37]. A recent report revealed that rituximab binds a discontinuous epitope in CD20 comprising four amino acids between A170 and S173 and five amino acids between Y182 and I186 [38]. Two amino acids in this region, C167 and C183, form a disulfide bond that appears critical for rituximab binding to maintain appropriate conformation of the epitope [38]. Initial studies indicated that CD20 density is a critical factor for rituximab-mediated CDC. However, it was recently shown by Teeling et al. that the binding characteristics and fine specificity of anti-CD20 mAbs also have a major influence on their ability to mediate CDC [33]. The fine specificity of mAbs recognizing a conformational epitope relies heavily on the target antigen's 3-D structure, which may be modulated by the plasma membrane. Disrupting the membrane of non-Hodgkin lymphoma (NHL) cells with detergents such as Triton X-100 results in loss of binding of anti-CD20 mAbs, again indicating that the topography of CD20 in the lipid bilayer is critical to epitope structure [33]. Altogether, accumulating evidence indicates that the ability of mAbs to interact with CD20 is dependent on both the expression level as well as steric restrictions imposed by its conformation in the plasma membrane. Our studies indicate that plasma membrane cholesterol is necessary for the maintenance of a CD20 conformational state that supports rituximab binding. By depleting cholesterol through inhibition of its synthesis with statins and berberine or through its direct removal with MβCD, we observed a significant reduction of rituximab-mediated CDC as well as binding of B9E9 to an extracellular loop of CD20. Cholesterol restitution experiments revealed even stronger rituximab-mediated CDC than in controls despite incomplete reversal of anti-CD20 staining. Exogenous cholesterol might play a dual role in this experimental setting: it might not only restore the conformation of CD20 (Figure 6), but also facilitate formation and penetration of the membrane attack complex into the plasma membrane. Similarly, erythrocytes for example have been shown to become more susceptible to lysis at increased cholesterol content [39].

The effect of statins did not result from shedding of CD20 from plasma membrane as has been shown for some other molecules. For example, CD30 was reported to localize to lipid rafts; after disruption with statins, MβCD, or filipin III, this molecule became exposed to membrane metalloproteases that generated soluble CD30 [40]. First, CD20 is recruited to lipid rafts only after anti-CD20 mAb binding, so it is unlikely that it is shielded from membrane metalloproteases [20,22]. Moreover, we could not restore binding of mAb to CD20 after co-incubation of Raji cells with lovastatin and phenantroline, a broad-specificity metalloprotease inhibitor (unpublished data). Restitution experiments revealed that a short 30-min incubation of Raji cells with exogenous cholesterol almost completely restores B9E9 binding as well as rituximab-mediated CDC in lovastatin-pretreated cells. Finally, immunofluorescence microscopy, labeling of extracellular proteins with sulfo-NHS-biotin, AFM, and the observation that maximal rituximab binding levels in flow cytometry are approximated at high antibody concentration clearly showed that CD20 is still present in plasma membrane of lovastatin-treated cells in levels comparable with that of untreated controls.

It can be argued that statins might also impair rituximab-mediated CDC by increasing the expression of complement-inhibitory proteins, including CD55 and CD59 [41,42]. However, this mechanism is unlikely to be responsible for statin-induced impairment of rituximab-mediated CDC, as we have not observed any induction of these proteins in Raji cells by Western blotting or flow cytometry (unpublished data).

Our knowledge on the influence of membrane cholesterol on the 3-D conformation of transmembrane proteins is rather limited. Cholesterol modulates the fluidity of biological membranes by inserting between fatty acid acyl chains, thereby abolishing phase transitions of bilayers [43]. Therefore, it is intuitive that the amount of membrane cholesterol might indirectly affect biophysical interactions between membrane lipids and proteins, especially in those that cross the plasma membrane several times. For example, MβCD-mediated cholesterol depletion from plasma membrane decreased the affinity of oxytocin to its receptor [44]. Cholesterol was also demonstrated to modulate the function of the cholecystokinin receptor through changes in membrane fluidity. Both receptors are G-protein coupled with seven transmembrane regions. Cholesterol in the endoplasmic reticulum causes conformational changes in SCAP, a polytopic endoplasmic reticulum membrane protein that cleaves sterol regulatory element binding proteins (SREBPs) [45]. Moreover, cholesterol depletion with MβCD was shown to interfere with antigen binding of the anti-CD20 mAb FMC7 [46] as well as several other CD20 mAbs [21].

We have observed that incubation of Raji cells with lovastatin renders CD20 resistant to ChT-limited proteolysis, thereby indicating that some critical ChT-sensitive residues become unavailable for digestion (Figure 7C and 7D). Moreover, AFM studies revealed that lovastatin-treated cells still express CD20, but the affinity (binding force) of rituximab is decreased, indicating potential conformational changes (Figure 7B). Also, a subtle difference in CD20 precipitation with sulfo-NHS-biotin (Figure 2J) might be caused by conformational changes in CD20, resulting in steric hindrance of some amino groups for biotinylation. Altogether, the data presented here indicate that statins appear to affect a cholesterol-dependent conformation of CD20, thereby impairing mAb binding, CDC, and ADCC of CD20-expressing lymphoma cells.

Critical issues for the clinical translation of our studies are the statin concentrations and accompanying changes in cholesterol levels achievable in patients. Serum statin concentrations in patients with hypercholesterolemia have been shown to be in sub-micromolar range. Nonetheless, in a recent study of lovastatin administered to patients with advanced cancers, serum lovastatin concentrations reached 12 μM [47]. In most of our studies we have used lovastatin and other statins at 10 μM concentration. At lower concentrations (2.5–5.0 μM), lovastatin also decreased rituximab-mediated CDC, indicating that the observed effects might be clinically relevant. Our in vitro studies, however, may not accurately reflect the clinical situation. Even though Raji cells were incubated with statins for only 48 h, and patients are taking cholesterol-reducing drugs on a regular basis for extended periods of time, it is quite possible that cholesterol is depleted more strongly in vitro than in vivo. Therefore, a small exploratory clinical study of patients with hypercholesterolemia was designed to test the hypothesis that in vivo changes in plasma cholesterol influence the binding of anti-CD20 antibodies to CD20-expressing cells. We observed that even a short-term atorvastatin treatment leads to a statistically significant reduction in anti-CD20 mAb binding, which roughly parallels the reduction in plasma cholesterol. The full impact of plasma cholesterol changes due to statin treatment (or other clinical, environmental, or genetic causes) on immunotherapy with rituximab and other anti-CD20 antibodies should be studied in clinical trials in which patients are stratified on basis of their plasma cholesterol content and/or statin usage.

The changes in cholesterol levels on CD20 epitope exposure, as demonstrated in the current study, have important clinical implications. It is possible that statins and potentially other cholesterol-lowering drugs can affect diagnosis of lymphoid malignancies, as often determined with antibody-based reagents on blood cells in flow cytometry. Fluctuations in plasma cholesterol may furthermore affect anti-CD20 therapy by decreasing activity under low-cholesterol conditions but increasing activity at high cholesterol. Consistency of anti-CD20 therapy might therefore benefit from the application of antibodies that are less sensitive to such changes and/or efficiently lyse cells at low antibody occupancy or low CD20 expression levels.

In summary, our study shows that changes in membrane cholesterol affect CD20 antibody binding in vitro and in vivo. The influence of statin treatment and plasma cholesterol on CD20 immunotherapy should be addressed in clinical studies with CD20 antibodies currently ongoing in oncology as well as in inflammatory diseases.

## Supporting Information

Alternative Language Abstract S1 Translation of the Abstract into Polish Translated by Jakub Golab.
(27 KB DOC)

## Abbreviations

ADCC: antibody-dependent cellular cytotoxicity
AFM: atomic force microscopy
CDC: complement-dependent cytotoxicity
ChT: chymotrypsin
FTI: farnesyltransferase
GGTI: geranylgeranyltransferase
HMG-CoAR: 3-hydroxy-3-methylglutaryl-coenzyme A reductase
MβCD: methyl-β-cyclodextrin
mAb: monoclonal antibody
MFI: mean fluorescence intensity
MTT: 3-[4,5-dimethylthiazol-2-yl]-2,5-diphenyltetrazolium bromide
pN: piconewton
T: trypsin
