# Supplementary material for: Statins Impair Antitumor Effects of Rituximab by Inducing Conformational Changes of CD20
Source: PLoS Med. 2008 Mar 25;5(3):e64. doi: 10.1371/journal.pmed.0050064 (PMC2270297; doi:10.1371/journal.pmed.0050064)
Supplement: Alternative Language Abstract S1 — Translated by Jakub Golab. (27 KB DOC) [file pmed.0050064.sd001.doc]

##### Streszczenie

**Przesłanki**: Rytuksymab jest stosowany w leczeniu CD20^+^ chłoniaków B-limfocytarnych oraz innych B-limfocytarnych zespołów limfoproliferacyjnych. Skuteczność kliniczną rytuksymabu można próbować zwiększać poprzez opracowanie terapii łączonych z innymi lekami, takimi jak statyny, które hamują syntezę cholesterolu i wykazują działania przeciwnowotworowe. Celem tej pracy było zbadanie wpływu statyn na cytotoksyczne działanie rytuksymabu względem chłoniaków B-limfocytarnych.

**Metody i Wyniki**: Nieoczekiwanie zaobserwowano, że statyny zmniejszają zdolność rytuksymabu do zabijania komórek chłoniaka przy udziale układu dopełniacza (CDC) i cytotoksyczności komórkowej zależnej od przeciwciał (ADCC). Cytotoksyczność badano przy użyciu testów MTT, Alamar blue oraz barwieniem błękitem trypanu, natomiast ADCC mierzone było przy użyciu testu z uwalnianiem ^51^Cr. Statyny zmniejszyły wiązanie przeciwciał anty-CD20 z powierzchnią komórek, ale nie wpływały na wytwarzanie cząsteczki CD20 w komórkach (brak wpływu w badaniach Western blotting oraz RT-PCR). Podobne efekty wywierały inne związki usuwające cholesterol z komórek (metylo- β -cyklodekstryna oraz berberyna), ale nie filipina III, co wskazywałoby na to, że obecność cholesterolu w błonie komórkowej, a nie obecność tratewek lipidowych jest niezbędna do działania rytuksymabu. W mikroskopii fluorescencyjnej zaobserwowano, że po inkubacji komórek nowotworowych ze statynami cząsteczka CD20 jest nadal zlokalizowana w błonie komórkowej, ale nie wiąże się z przeciwciałami skierowanymi przeciw jej epitopom nieliniowym. Wiązanie do epitopów liniowych pozostaje nie zmienione. Przy użyciu mikroskopii sił atomowych oraz ograniczonej proteolizy ustalono, że usunięcie cholesterolu z błony komórkowej doprowadza do zmian konformacyjnych cząsteczki CD20, co osłabia wiązanie z nią przeciwciał. Zmniejszenie stężenia cholesterolu uzyskane w wyniku leczenia atorwastatyną 5 pacjentów z hipercholesterolemią zmniejszyło wiązanie przeciwciał anty-CD20 z powierzchnią limfocytów B.

**Wnioski**: Statyny upośledzają zarówno wiązanie się z komórkami jak i aktywność przeciwnowotworową rytuksymabu. Obserwacje te maja ważne implikacje kliniczne bowiem indukowane przez statyny osłabienie wiązania przeciwciał konformacyjnych z cząsteczką CD20 na powierzchni komórek nowotworowych może opóźnić zarówno rozpoznanie i rozpoczęcie leczenia jak i zmniejszyć skuteczność terapeutyczną rytuksymabu.
